# The MYPT2-regulated striated muscle-specific myosin light chain phosphatase limits cardiac myosin phosphorylation *in vivo*

**DOI:** 10.1016/j.jbc.2024.105652

**Published:** 2024-01-13

**Authors:** Eunyoung Lee, Herman May, Katarzyna Kazmierczak, Jingsheng Liang, Nhu Nguyen, Joseph A. Hill, Thomas G. Gillette, Danuta Szczesna-Cordary, Audrey N. Chang

**Affiliations:** 1 Department of Internal Medicine, University of Texas Southwestern Medical Center, Dallas, Texas, USA; 2 Department of Molecular and Cellular Pharmacology, University of Miami Miller School of Medicine, Miami, Florida, USA; 3 Pak Center for Mineral Metabolism and Clinical Research, UTSW Medical Center, Dallas, Texas, USA

**Keywords:** cardiac muscle, MLCP, myosin, protein phosphorylation, protein phosphatase, enzyme, MYPT2

## Abstract

The physiological importance of cardiac myosin regulatory light chain (RLC) phosphorylation by its dedicated cardiac myosin light chain kinase has been established in both humans and mice. Constitutive RLC-phosphorylation, regulated by the balanced activities of cardiac myosin light chain kinase and myosin light chain phosphatase (MLCP), is fundamental to the biochemical and physiological properties of myofilaments. However, limited information is available on cardiac MLCP. In this study, we hypothesized that the striated muscle-specific MLCP regulatory subunit, MYPT2, targets the phosphatase catalytic subunit to cardiac myosin, contributing to the maintenance of cardiac function *in vivo* through the regulation of RLC-phosphorylation. To test this hypothesis, we generated a floxed-PPP1R12B mouse model crossed with a cardiac-specific Mer-Cre-Mer to conditionally ablate MYPT2 in adult cardiomyocytes. Immunofluorescence microscopy using the gene-ablated tissue as a control confirmed the localization of MYPT2 to regions where it overlaps with a subset of RLC. Biochemical analysis revealed an increase in RLC-phosphorylation *in vivo*. The loss of MYPT2 demonstrated significant protection against pressure overload-induced hypertrophy, as evidenced by heart weight, qPCR of hypertrophy-associated genes, measurements of myocyte diameters, and expression of β-MHC protein. Furthermore, mantATP chase assays revealed an increased ratio of myosin heads distributed to the interfilament space in MYPT2-ablated heart muscle fibers, confirming that RLC-phosphorylation regulated by MLCP, enhances cardiac performance *in vivo*. Our findings establish MYPT2 as the regulatory subunit of cardiac MLCP, distinct from the ubiquitously expressed canonical smooth muscle MLCP. Targeting MYPT2 to increase cardiac RLC-phosphorylation *in vivo* may improve baseline cardiac performance, thereby attenuating pathological hypertrophy.

The phosphorylation of cardiac myosin at its regulatory light chain (RLC) subunit is a modulatory post-translational modification that, while not required for cardiac development or contractile force generation, was shown to be necessary for normal cardiac function through genetic targeting of its dedicated cardiac myosin light chain kinase (cMLCK) (1, 2, 3, 4). Human hearts in failure have lower cMLCK expression and, thus, lower RLC-phosphorylation (4, 5). *In vitro*, cardiac RLC-phosphorylation causes increased Ca^2+^ sensitivity of cardiac myofilament contraction, generating faster and higher isometric forces in isolated muscle strips (6, 7). Based on evidence that many hypertrophic cardiomyopathy-associated mutations cause increased Ca^2+^ sensitivity of myofilament contractions (8), cardiac hypertrophy is predicted to develop in animals with elevated baseline constitutive cardiac RLC-phosphorylation. However, contrary to hypertrophy observed in animal models and humans with hypertrophic cardiomyopathic mutations, increased baseline levels of RLC-phosphorylation from 0.4 to 0.6 mol phosphate/mol RLC was shown to attenuate maladaptive hypertrophic responses in two different transgenic mouse models (1, 9). Thus, RLC-phosphorylation is a property that is fundamental to the determination of cardiac function and malfunction. Despite compelling studies that point toward therapeutic targeting of RLC-phosphorylation, it is not known whether the attenuation of hypertrophy observed in model systems is due to the overexpression of the MLCK transgenes, and the specific contributions of the cardiac myosin light chain phosphatase (MLCP) toward the anti-hypertrophic phenotype remain understudied.

Conventional type II myosins, which include muscle and nonmuscle myosins, are ATPases comprised of two myosin heavy chains, two essential light chains, and two RLCs (10). The regulation of myosin ATPase activity is best characterized in smooth muscle and nonmuscle cells, where phosphorylation of the RLC induces required structural changes in the myosin molecule (11, 12). Smooth muscle and nonmuscle myosins are activated by Ca^2+^/calmodulin-dependent smooth muscle MLCK to induce myofilament shortening and, thereby, muscle contraction (13, 14). Relaxation of contracted smooth muscles is dependent on dephosphorylation of the RLC by smooth muscle MLCP, which is a trimeric holoenzyme comprised of a regulatory myosin phosphatase target subunit 1 (MYPT1), a catalytic type 1 phosphatase subunit (PP1cβ), and an accessory protein (M20) (15, 16). The ubiquitously expressed smooth muscle MLCP is regulated by numerous mechanisms through MYPT1 and is widely studied in nonmuscle and smooth muscle cells (16, 17), but its specific role in striated muscles is unknown.

Constitutive RLC-phosphorylation in beating hearts is maintained by constitutive activities of cMLCK in balance with opposing MLCP activities (18, 19). In striated muscles, myosin phosphorylation is not required for contractions, but studies of various animal models support that fine-tuning of myosin activity is necessary to adapt to physiological and pathological conditions (20, 21, 22). MYPT2 is the dominant phosphatase regulatory subunit expressed, but there is limited information on MYPT2 localization and contributions to regulating cardiac RLC-phosphorylation in intact systems. MYPT2-PP1cβ dephosphorylates intact myosin at its RLC *in vitro* (23), implying its role as a MLCP *in vivo*. The C-terminal regions of the gene for MYPT2, PPP1R12B, encodes for M20, which is the smooth muscle accessory protein that together with MYPT1 and PP1cβ forms the smooth muscle MLCP holoenzyme (24). Thus, MLCP isolated from skeletal muscle extracts does not have M20 because MYPT2 is expressed instead (24).

Overexpression of MYPT2 in the heart led to a small decrease in cardiac myosin phosphorylation, presumably due to the accumulation of PP1cβ. However, immunogold labeling showed localization of MYPT2 at the Z-disc, where myosin is not located (23). In snap-frozen mouse hearts, processed to preserve phosphorylations, MYPT2 is maximally phosphorylated, indicating that cardiac MLCP activity is completely inhibited (19). It is not known whether maximal MYPT2 phosphorylation is a result of responses to elevated sympathetic tone in the mice undergoing euthanasia or if MYPT2 has other roles in the cardiac myocyte that is unrelated to the modulation of myosin phosphorylation.

Knockout of the canonical MLCP regulatory subunit MYPT1 causes death during embryonic development (25). MYPT1 is ubiquitously expressed and implicated in the determination of cell polarity in cytokinesis (26), regulation of cell growth through YAP-mediated signaling (27), and cell motility (28). MYPT2 has 87% sequence homology with MYPT1, with conservation of all major structural domains. Recent publications in cancer and reports of human polymorphisms suggest that, like MYPT1, MYPT2 may have diverse roles (29, 30, 31, 32, 33). However, many of these studies were not validated at the protein level, and the specificity of the antibodies used is uncertain.

In order to improve basic understanding of MYPT2 as it pertains to cardiac muscle biology, we generated a floxed-PPP1R12B mouse model to circumvent potential embryonic lethality and developmental compensatory mechanisms and conditionally knocked out MYPT2 protein in the adult mouse cardiac myocytes *in vivo*. Using pan-MYPT and specific MYPT2 antibodies, we defined its localization within the cardiac myocyte, measured the effect of gene ablation on other regulators of myosin RLC-phosphorylation, examined the stability of a sequestered, super-relaxed (SRX) state of myosin and the number of heads accessible to actin, and determined its contributions to cardiac function *in vivo*. The studies reported herein establish the fundamental properties of the cardiac muscle MLCP. Cardiac muscle MLCP that MYPT2 regulates directly limits RLC-phosphorylation to affect the number of myosin heads in the disordered relaxed (DRX) state. Reduction in MLCP activity increases the baseline availability of unsequestered myosins, directly enhancing cardiac functional reserve.

## Results

### Confirmation of targeted cardiac MYPT2 protein ablation and effects on MYPT1

**Figure 1:**
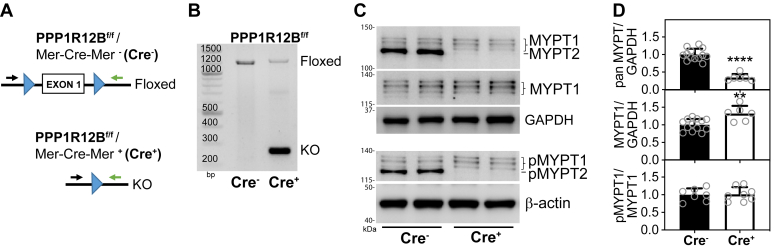
**Confirmation of tamoxifen-induced gene ablation in PPP1R12B**^**f/f**^**/Cre+ mice**. *A*, scheme illustration of lox-P guided generation of the knockout allele. *Triangles* represent Lox-P sites, *arrows* represent approximate locations of forward and reverse primers used in confirming presence of the floxed and knockout alleles by PCR. *Upper cartoon* depicts the control mouse where the exon 1 of the gene that encodes MYPT2 protein, PPP1R12B (PPP1R12B^f/f^), is floxed but is negative for the Mer-Cre-Mer transgene (Cre-). *Lower cartoon* depicts the KO mouse where exon 1 has been spliced out by the Mer-Cre-Mer that it was positive for (Cre+). *B*, representative gel image of DNA segments amplified using primers illustrated in *panel A*, from indicated mouse ventricle DNA extracts collected after tamoxifen treatment. *C*, representative immunoblots of total ventricular tissue extracts for proteins indicated using specific antibodies; MYPT2 was undetectable in samples from Cre+ animals. *D*, quantification of total MYPT and inhibited MYPT1 (pMYPT1/MYPT1), that shows total MYPT1 is increased in the PPP1R12B^f/f^/Cre+ mice after tamoxifen treatment, but the ratio of activated MYPT1 is not. ∗∗∗∗*p* < 0.0001, ∗∗<0.01 by unpaired *t* test, two-tailed, GraphPad Prism. MYPT1, myosin phosphatase target subunit 1.

We generated a mouse line to conditionally knockout the PPP1R12B gene to reduce MYPT2 protein in targeted tissues. Modeled after a previously published MYPT1 targeting strategy (34), lox-P sites were inserted flanking exon 1 of PPP1R12B and confirmed by sequencing. Mice with the floxed allele were crossed with Mer-Cre-Mer transgenic mouse line to conditionally knockout exon 1 and reduce MYPT2 protein in adult cardiac myocytes. A simplified schematic of the floxed and KO allele is shown (Fig. 1*A*). Primers directed at regions outside of exon 1 and lox-P sites were used to confirm genetic ablation of the sequence between lox-P by PCR. PCR of total DNA from ventricles collected from mice 2 weeks after tamoxifen injections shows the KO band is detectable only in PPP1R12B^f/f^/Cre+ mice (Fig. 1*B*). MYPT2 protein in mouse ventricular tissue extracts was undetectable by immunoblots in PPP1R12B^f/f^/Cre+ animals (Fig. 1*C*), despite the presence of heterogeneous cell types in cardiac muscles. This suggests that MYPT2 protein expression is normally most abundant in cardiac myocytes. A pan antibody was used to detect MYPT2 in relation to MYPT1 in order to obtain information on stoichiometry. Due to the size difference between MYPT1 and MYPT2, there is no overlap in protein detection using the pan antibody. When quantified using a pan-MYPT antibody, we measured that total MYPTs in the targeted samples (Cre+) were reduced to 34% of WT (Cre-), indicating that MYPT2 accounts for greater than two-thirds of total MYPT1+2 (Fig. 1*D*, top panel). The 66% reduction in total MYPT1+2 in the PPP1R12B^f/f^/Cre+ includes a 32% increase in MYPT1 expression (Fig. 1*D*, middle panel). However, since total MYPT1 accounts for only one-third of total MYPT1+2, this increase compensates for about 10% of the total (Fig. 1*D*). Phosphorylation of MYPT1 at Thr694 (Thr696 in human sequence) inhibits MLCP activity *in vivo* (35). We asked whether MYPT1 was significantly dephosphorylated at this residue and thereby activated in PPP1R12B^f/f^/Cre+ hearts to compensate for the large reduction in baseline MLCP activity. Total MYPT1-normalized phosphorylated MYPT1 levels were not significantly different between the control and MYPT2.

### Localization of MYPT2 in the mouse heart

**Figure 2:**
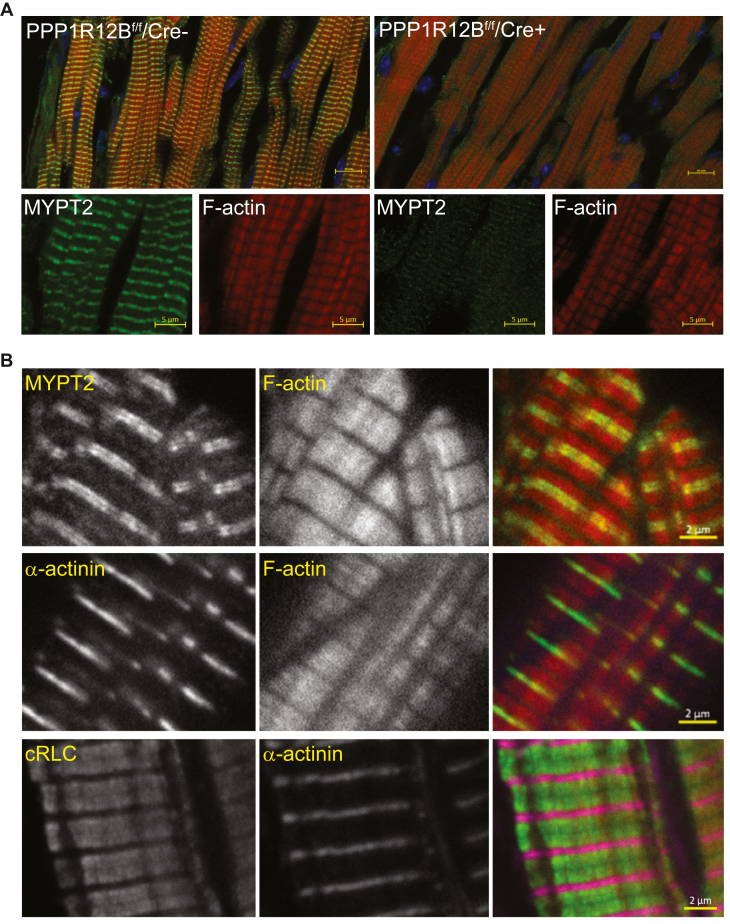
**Immunofluorescence images of the left ventricular free wall, stained as indicated.** Hearts from male 9 to 12 week old PPP1R12B^f/f^/Cre- and PPP1R12B^f/f^/Cre+ mice, 3 weeks after tamoxifen-treatment were perfusion-fixed and cryosectioned as detailed in methods. *A*, representative upper merged image shows MYPT2 is present in regions that overlap phalloidin stained actin in PPP1R12B^f/f^/Cre-sarcomeres to a greater degree than in PPP1R12B^f/f^/Cre+ (Scale bar- 10 μm). Enlarged selected regions below merged images show MYPT2 is undetectable in PPP1R12B^f/f^/Cre+ sections (Scale bar- 5 μm). *B*, representative higher magnification images of MYPT2 (*top row*), α-actinin (*middle row*), and cardiac RLC (*bottom row*) localization, relative to indicated costained structures shown in adjacent panels (*center column*) and merged composite images (*last column*). Composite image of cRLC and α-actinin shows that at this resolution, regions that correspond to the I-band or D-zone of the myofilament are not discernable. RLC spans across the whole sarcomere immediately adjacent to Z-disc region identified by α-actinin staining. MYPT2 stains two distinct regions apparently on either side of α-actinin, where it may partly overlap with RLC. RLC, regulatory light chain.

Localization of MYPTs in the heart by immunoelectron microscopy previously showed accumulation in the Z-disk, where myosin RLC is not present (23). Using hearts from tamoxifen-treated PPP1R12B^f/f^/Cre+ mice as a negative control, we documented the expression and distribution of MYPT2 in cardiac muscle by immunofluorescence (Fig. 2). MYPT2 protein signal was the highest in F-actin-positive muscle layers of the heart where cardiac muscle myosins are found, confirming its localization near RLC. To further localize the expression of MYPT2 along the sarcomere, we stained triton-washed mouse cardiac myofibrils with α-actinin, MYPT2, or cardiac RLC (Fig. 2*B*). Compared to α-actinin, MYPT2 appeared to localize in regions adjacent to the Z-disc. At this resolution, the localization of MYPT2 to the I-band or D-zone is not discernable but likely is excluded from the C-zone where myosin binding protein C is localized (36). RLC localization that covers the whole span of the sarcomere, adjacent to α-actinin, confirms MYPT2 partly overlaps with a subset of RLC.

### Effects of MYPT2 protein ablation on RLC-phosphorylation and heart size

**Figure 3:**
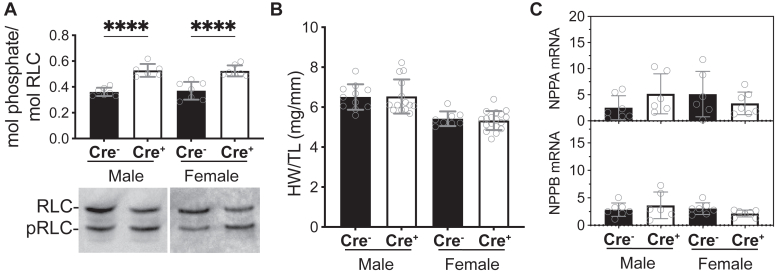
**Effects of PPP1R12B targeted gene ablation on male and female hearts**. *A*, baseline cardiac RLC-phosphorylation, determined as mol phosphate/mol RLC. Molar extents of phosphorylation was calculated from the ratio of phosphorylated (pRLC)/total (RLC + pRLC). Shown below quantification bars are representative immunoblot of RLC after separation of pRLC from nonphosphorylated RLC by glycerol-PAGE. Corresponding ventricular extracts from male and female mice, 2 to 3 weeks after tamoxifen treatment are shown. *B*, baseline heart size, as determined by measurements of whole heart wet tissue weight/mouse tibial length, *C*, qRT-PCR delta Ct values of cardiac hypertrophy-associated genes that encode ANP (NPPA) and BNP (NPPB), relative to 18S. *Open circles* represent individual mice; N ≥ 6 for all bars in figure, ∗∗∗∗*p* < 0.0001 by ordinary two-way ANOVA, followed by Tukey’s multiple comparisons test using GraphPad Prism. RLC, regulatory light chain.

Given that the extent of cardiac RLC-phosphorylation is determined by balanced activities of the kinase and phosphatases that act on it, we first asked how cardiac RLC-phosphorylation was affected in the hearts where MYPT2 protein was removed. Two to 4 weeks after tamoxifen treatment, cardiac RLC-phosphorylation was significantly increased in the PPP1R12Bf/f/Cre+ mice hearts by 46%, from around 0.37 in the WT to 0.53 mol phosphate/mol RLC in the KO (Fig. 3*A*). The increased RLC-phosphorylation was similar in male and female mice. Numerous mouse models of hypertrophic cardiomyopathy show an increase in cardiac muscle mass that is induced, in part, by increased Ca^2+^ sensitivity of contractile force (8). Biochemical studies have shown phosphorylation of cardiac RLC directly causes increased Ca^2+^ sensitivity of myosin activity and maximal isometric force (37, 38). In this model, we asked whether an increase in baseline RLC-phosphorylation also causes cardiac hypertrophy. Comparison of mouse heart weight/tibial length ratios showed no difference in heart sizes in tamoxifen-treated PPP1R12B^f/f^/Cre- or PPP1R12B^f/f^/Cre+ mice of either sex (Fig. 3*B*), suggesting no phosphorylation-induced cardiac growth. Consistent with heart size comparisons, quantitative RT-PCR of hypertrophy markers showed no differences in baseline levels of heart growth-associated genes (Fig. 3*C*), indicating that hypertrophy-related gene transcription is not activated in caged sedentary mice, despite a significant increase in RLC-phosphorylation.

### Compensatory changes in regulators of RLC-phosphorylation in response to MYPT2 protein ablation

**Figure 4:**
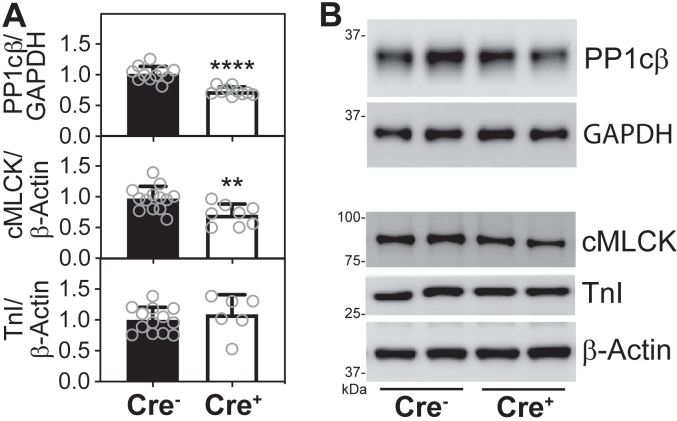
**Effects of MYPT2 protein ablation on other RLC-phosphorylation regulators.***A* and *B*, quantification (*A*) and representative immunoblots (*B*) of proteins indicated, in PPP1R12B^f/f^/Cre- and PPP1R12B^f/f^/Cre+ mice ventricular extracts after tamoxifen treatment. Combined comparison of three different cohorts relative to respective average value in Cre-group; *open circles* represent individual mice, N ≥ 6 for all bars in figure, ∗∗∗∗*p* < 0.0001, ∗∗<0.01 by unpaired *t* test, two-tailed, GraphPad Prism. RLC, regulatory light chain.

We previously reported that targeting MYPT1 or PP1cβ in smooth muscles *in vivo* caused a significant reduction in the expression level of the other protein, which suggests that the proteins of the MLCP holoenzyme stabilized one another (39, 40). Unlike in smooth muscles, targeted knockdown of PP1cβ in cardiac muscle did not cause a comparable reduction in MYPT1 or MYPT2 (41). We then measured the effects of MYPT2 protein ablation on the expression of PP1cβ in the hearts of mice. We discovered that PP1cβ was significantly reduced (Fig. 4, *A* and *B*, top), similar to levels when MYPT1 is completely ablated in smooth muscles (40). In addition, baseline cMLCK expression was reduced by 27% in the hearts of tamoxifen-treated PPP1R12B^f/f^/Cre+ mice (Fig. 4, *A* and *B*, middle). Cardiac muscle-specific troponin I expression was unchanged, confirming that thin filament proteins are unaffected by the reduction in myosin-targeting subunit MYPT2 (Fig. 4, *A* and *B*, bottom). Thus, reduction in both cMLCK and MYPT2 proteins in the heart contributed to the increased baseline cardiac RLC-phosphorylation.

### PPP1R12B^f/f^/Cre+ animals have reduced hypertrophic response to pressure overload

**Figure 5:**
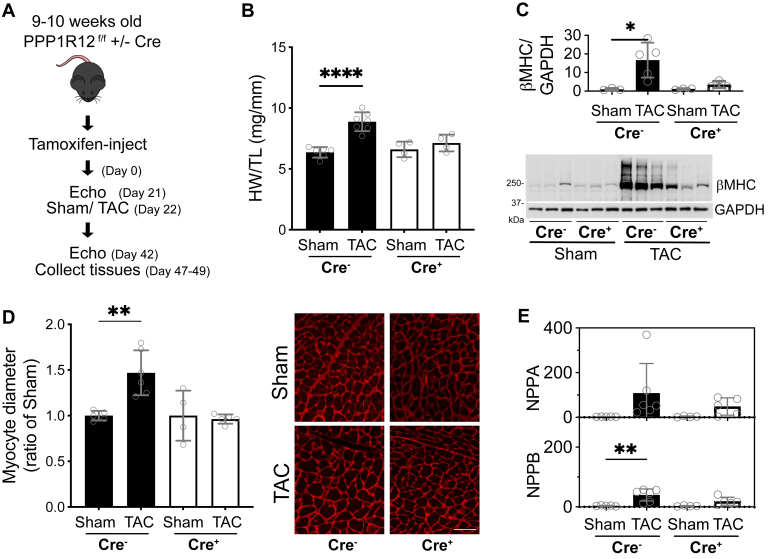
**Effects of applied pressure overload induced cardiac hypertrophy**. *A*, scheme of applied transaortic constriction (TAC) stress on PPP1R12B^f/f^/Cre- and PPP1R12B^f/f^/Cre+ male mice. *B*, heart size comparison after Sham or TAC surgeries, shown as heart weight/tibial length (HW/TL). *C*, quantification and representative immunoblot of heart extracts for β-myosin heavy chain expression. *D*, myocyte size comparison, determined by cross-sectional area measurements of wheat germ agglutinin–stained heart sections. Representative images are shown, scale bar represents 50 μm. *E*, quantitative RT-PCR of NPPA and NPPB in samples indicated. For all quantification bars, *open circles* represent individual mice; N ≥ 4 for all bars in figure, ∗∗∗∗*p* < 0.0001, ∗∗*p* < 0.01, ∗*p* < 0.05 by ordinary two-way ANOVA, followed by Tukey’s multiple comparisons test using GraphPad Prism.

Previous publications by others have shown that transgenic animals with increased MLCK expression in the myocyte are protected from pathological hypertrophy induced by isoproterenol infusion or pressure overload (1, 9). We tested the hypothesis that those observed attenuated responses were a direct result of increased baseline cardiac RLC-phosphorylation and not due to increased MLCK transgene expression. PPP1R12B^f/f^/Cre- and PPP1R12B^f/f^/Cre+ animals were treated with tamoxifen and, after at least 3 weeks of recovery, subjected to pressure overload by transaortic constriction (TAC) for 3 to 4 weeks. Physiological effects were documented by echocardiography before surgery and 3 weeks after, as shown in the schematic (Fig. 5*A*). TAC surgery did not induce significant differences in cardiac parameters measured by echocardiography within the 3-week timepoint (Table S1). In contrast, a comparison of heart weight to tibia length ratios of animals subject to Sham operation and TAC shows that while control PPP1R12B^f/f^/Cre-animals have significant cardiac hypertrophy after TAC, the PPP1R12B^f/f^/Cre+ hearts show no evidence of hypertrophic growth (Fig. 5*B*). These observations were confirmed by measurements of β-myosin heavy chain protein expression (Fig. 5*C*) (42) and myocyte diameter in wheat germ agglutinin (WGA)-stained heart sections, which demonstrated increased diameters in response to TAC in the control mice but not in the PPP1R12B^f/f^/Cre+ mice (Fig. 5*D*). Transcriptomic signatures of hypertrophy, NPPA and NPPB, were also blunted in the PPP1R12B^f/f^/Cre+ TAC surgery group when compared to the WT response (Fig. 5*E*). These data are consistent with a model that MYPT2-regulated MLCP activity limits RLC-phosphorylation *in vivo*, and targeting of MLCP enhances cardiac performance reserve.

### PPP1R12B^f/f^/Cre+ animals have normal protein responses to pressure overload

**Figure 6:**
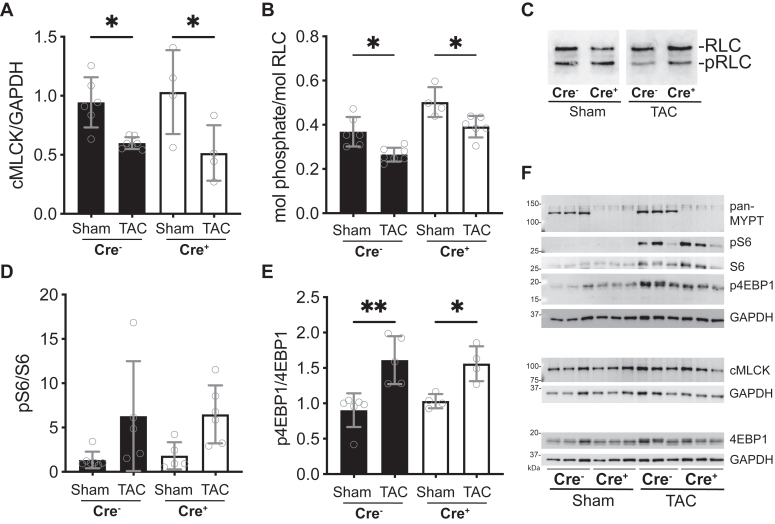
**Effects of pressure overload on proteins predicted to be differentially regulated**. *A*, comparisons of cardiac MLCK expression. *B*,*C*, quantified extents of cardiac RLC-phosphorylation (*B*) representative immunoblot of RLC after separation of phosphorylated (pRLC) from unphosphorylated RLC (*C*). *D* and *E*, quantification of mTOR downstream effector pS6/S6 (*D*) and p4EBP1/4EBP1 (*E*). *F*, representative immunoblots. GAPDH panel below 4EBP1 is the same panel shown in Figure 5*C*, where the upper portion of the blot was probed for β-myosin heavy chain expression, and the lower panel probed for total 4EBP1 and GAPDH. For all quantification bars in figure, *open circles* represent individual mice; N ≥ 4 for all bars in figure, ∗∗*p* < 0.01, ∗*p* < 0.05 by ordinary two-way ANOVA, followed by Tukey’s multiple comparisons test using GraphPad Prism. MLCK, myosin light chain kinase; RLC, regulatory light chain.

Based on the significant attenuation of TAC-induced cardiac hypertrophy in the MYPT2 knockout mice, we asked whether known protein expression responses to TAC surgery were dysregulated in the MYPT2 knockout animals. Consistent with previous reports by others (1), we measured that applied pressure overload caused a significant reduction in cMLCK expression (Fig. 6*A*) and 0.1 mol phosphate/mol reduction in RLC-phosphorylation compared to sham in both PPP1R12B^f/f^/Cre- and PPP1R12B^f/f^/Cre+ animals (Fig. 6*B*). In the presence or absence of MYPT2 protein, the amount of cMLCK reflects the extent of RLC-phosphorylation, confirming that cMLCK expression is a major determinant of RLC-phosphorylation.

Increased expression of mTOR signaling axis proteins in response to TAC-induced pressure overload is well documented (43). Based on blunted hypertrophy of PPP1R12B^f/f^/Cre+ animal hearts, we asked whether downstream effectors of mTOR, 4EBP1, and S6 were similarly activated in response to TAC. Consistent with the reduction in RLC-phosphorylation with TAC-induced stress, mTOR effectors were significantly activated in both Cre- and Cre+ groups (Fig. 6, *D*–*F*). MYPT2 knockout hearts had similar levels of mTOR-related signaling increase, consistent with decreases in cMLCK expression and RLC-phosphorylation. Thus, TAC-induced hypertrophy signaling is similarly activated in MYPT2 knockout animals, but no increase in heart mass was observed compared with PPP1R12B^f/f^/Cre-animals. These results suggest that increased muscle contractile demand imposed by pressure-overload is sufficiently met in the PPP1R12B^f/f^/Cre+ animals.

### The ratio of DRX/SRX in PPP1R12B^f/f^/Cre+ animals is greater than in PPP1R12B^f/f^/Cre- control

Phosphorylation of cardiac RLC directly modulates the Ca^2+^-sensitivity of force development and the kinetics of cycling myosin cross-bridges (44, 45) through its structural effects on the switch between the OFF and ON conformations of myosin motors and a movement of myosin heads toward the thin filaments (46, 47). The evidence gathered from phenotyping the MYPT2 targeted hearts points to attenuation of hypertrophic response through retention of cardiac RLC-phosphorylation at the normal level of 0.4 mol phosphate/mol RLC. These observations suggest a myosin activity-dependent performance threshold exists in the heart that accommodates changes in hemodynamic load. Post tamoxifen treatment, we sought to determine, through biomolecular characterization of the PPP1R12B^f/f^/Cre+ and PPP1R12B^f/f^/Cre- mouse heart fibers, direct evidence that the myosin molecules indeed had a greater performance capacity.

Myosin molecules undergo physiological transitions between the SRX state, maximizing energy conservation, and the DRX state, enabling cross-bridge formation with greater ATP consumption (48). In the DRX state, myosin heads are readily available to interact with actin, producing force and enhancing cardiomyocyte contractility. Under normal conditions, these two myosin conformations are in energetic equilibrium, crucial for maintaining normal heart function. Destabilization of the myosin energy conserving SRX state can trigger contractile abnormalities, morphological and metabolic remodeling, and result in adverse clinical outcomes (49).

**Figure 7:**
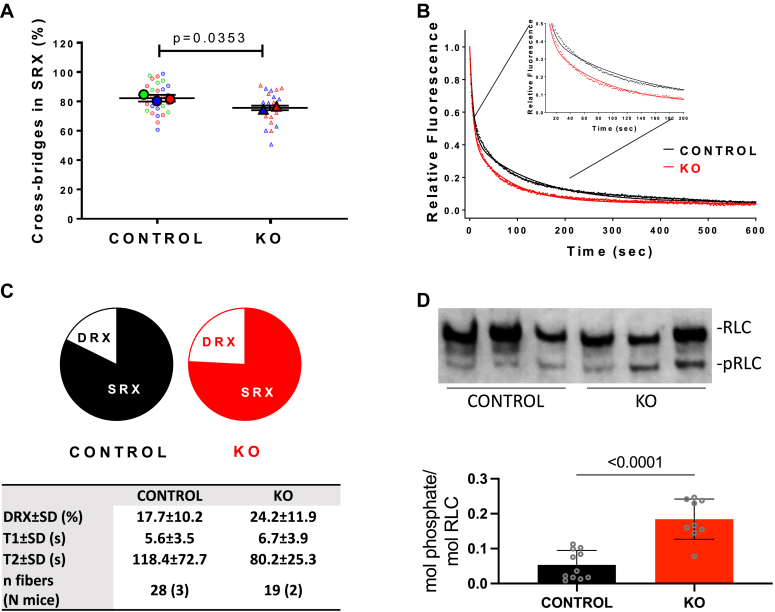
**Regulation of the super-relaxed (SRX) state of myosin in LVPM fibers from MYPT2 ablated hearts**. *A*, SuperPlots depicting cross-bridges occupying the SRX state in PPP1R12B^f/f^/Cre- (CONTROL) and PPP1R12B^f/f^/Cre+ (KO) mice. Data per animal (N) are presented using large, color-coded symbols, while respective fiber (n) measurements are indicated by small, color-coded symbols. Errors are represented as standard deviation and calculated per animal (12-week-old male mice, 3 weeks post-tamoxifen treatment). *B*, fluorescence decay curves over time of mantATP release chased with excess dark ATP. *C*, distribution of myosin heads (in %) between the disordered relaxed (DRX) and SRX states, along with their respective lifetimes (in seconds) in Control *versus* KO mice. *D*, representative immunoblot and quantification of single fibers (n ≥ 9) from Control and KO mice after DRX/SRX measurements. All mice used were males to maintain consistency with surgery groups, where only males were utilized, as discussed in the Methods section. Significance was determined by unpaired *t* test, two-tailed, using GraphPad Prism. mant, N-methylanthraniloyl.

To elucidate the effect of MYPT2 gene ablation on myosin energetics and the SRX↔DRX equilibrium, we isolated left ventricular papillary muscle (LVPM) fibers from PPP1R12B^f/f^/Cre+ (KO) and PPP1R12B^f/f^/Cre- (CONTROL) mouse hearts and subjected them to N-methylanthraniloyl (mant)ATP chase experiments as described previously (50, 51, 52). A significantly lower proportion of myosin cross-bridges in the SRX state was observed for fibers from KO *versus* CONTROL mice (Fig. 7, *A*–*C*), suggesting that MYPT2 gene ablation causes the destabilization of myosin SRX conformation in these mice. The extent of RLC-phosphorylation after the experiments, given as mol phosphate/mol RLC and quantified from the ratio of phosphorylated RLC to the sum of nonphosphorylated and phosphorylated RLC, was 0.05 ± 0.04 in CONTROL and 0.18 ± 0.02 in KO mice (Fig. 7*D*). Although RLC-phosphorylation was significantly reduced in both WT and KO sample sets during the fiber isolation process, the increase in the DRX heads coincided with higher RLC-phosphorylation observed in PPP1R12B^f/f^/Cre+ animals (Fig. 7*D*). There were no statistical differences in lifetimes (in seconds) of the DRX (T1) and SRX (T2) states between the two genotypes (Fig. 7, *B* and *C*). These results suggest that increased RLC-phosphorylation in PPP1R12B^f/f^/Cre+ mice prompts myosin cross-bridges to enter the DRX state to interact with thin filaments and produce force and muscle contraction, the phenomenon previously observed in pseudo-phosphorylated RLC (S15D-RLC) reconstituted LVPM from cardiomyopathy mice (51, 52).

## Discussion

MYPT2 has been implicated in biochemical studies as the main regulatory protein for cardiac MLCP. Here we provide definitive proof of this observation through genetic ablation of MYPT2 in adult cardiomyocytes, resulting in a decrease in MLCP activity. Furthermore, we demonstrate a direct relationship between increased endogenous cardiac RLC-phosphorylation and a blunted hypertrophic response to mild pressure overload. Finally, we observe a higher ratio of myosin heads in the DRX state in the MYPT2-targeted hearts. Together, these findings suggest a model in which a performance threshold, determined by the extent of RLC-phosphorylation, regulates compensatory muscle growth independent of hemodynamic pressures. Indeed, while TAC leads to a decrease in RLC-phosphorylation, the MYPT2 gene-ablated animals under TAC conditions display a level of RLC-phosphorylation similar to that found at baseline in a WT heart. This suggests under conditions where the usual numbers of myosins are available to contribute to contractions, mild pressure overload is insufficient to induce compensatory growth (as there is nothing to compensate for). This is consistent with the attenuation of pathological and physiological hypertrophy in other models where RLC kinases were overexpressed in mice (1, 9).

Destabilization of the myosin energy-conserving SRX state is associated with contractile abnormalities, morphological and metabolic remodeling, and adverse clinical outcomes in patients with hypertrophic cardiomyopathy caused by mutations in the myosin heavy chain (53), myosin regulatory (50) and essential (54, 55) light chains. However, when RLC-phosphorylation is directly targeted *in vivo*, either by overexpression of MLCK (1, 9), knockout of the MLCP (herein), or expression of a RLC phosphomimetic (52, 56, 57), destabilization of SRX state does not exacerbate but appears to reduce hypertrophic responses. Increased heart size or weight is not a prognostic phenotype in these animals, contrary to HCM mutation-harboring mouse models. A potential partial explanation for the discrepancy lies in the contributions of distinct pools of activated myosins across the sarcomere. HCM mutations in the myosin heavy chain would affect every myosin molecule in the sarcomere, irrespective of the location of other regulators along the myofilament. Based on our immunofluorescence images of MYPT2 localization, which we confirmed as specific using MYPT2 KO tissue, RLC-phosphorylation regulation by MLCP may be more dynamic outside of the C-zone where MYBP-C is not present. It is not clear how the localization of MYPT2 balances out the localization of the kinases for RLC, which are all known to be diffusely expressed throughout the cytoplasm. However, this interpretation based on MYPT2 localization supports a broader range of fine-tuning of muscle performance modulation through RLC-phosphorylation. Moreover, RLC-phosphorylation in distinct areas of the myofilament may have additional roles other than modulation of the SRX/DRX state of the myosin molecules.

In context of the extent of RLC-phosphorylation *in vivo*, which only goes up to ∼65% in cardiac myocytes when the MLCK is overexpressed and the specific activity is increased by orders of magnitude (1, 9), fine-tuning of cardiac performance through coordination with other modulatory mechanisms is consistent with our measurements of electrically paced intact muscle strips, isolated intact myosin filaments, and washed myofibrils. These structures could be phosphorylated to greater than 85% *via* a first order phosphorylation mechanism only in the presence of nonspecific phosphatase inhibitors (19). Careful comparisons of various models are needed to ascertain whether RLC-phosphorylation distribution across the sarcomere is even *in vivo* and whether inotropic mechanisms differentially regulate distinct pools of RLC in relation to other myofilament proteins that are post-translationally regulated. These types of studies are currently limited due to sympathetic tone-induced hyperphosphorylation of proteins in animals undergoing euthanasia, rapid dephosphorylation of endogenous regulatory proteins in tissues after excision, and the lack of careful quantification of extents of various post-translational modifications that elicit physiologically relevant functional changes *in vivo*.

Major limitations of our current studies are that hearts were dissected 1 week after echocardiography measurements, which were performed at only one time point, 3 weeks after pressure-overload, to evaluate cardiac function. Thus, significant increases in heart weight and canonical protein and mRNA hypertrophy markers that were measured at 4 weeks after aortic banding were not captured by echocardiographic measurements conducted 1 week prior. Additionally, longitudinal studies that span 1 to 16+ weeks after pressure-overload could have provided greater insight into the role of ablating MYPT2 in pathological hypertrophy and heart failure. However, based on previous work by others who targeted RLC-phosphorylation increase through overexpression of MLCK in the heart, our limited studies may not have missed much in terms of remodeling processes attributed to increased RLC-phosphorylation. In a transgenic animal where cardiac RLC-phosphorylation is constitutively elevated, heart size measured by heart weight/tibial length was not increased from 2 to 6 months of age (9), suggesting that predicted sustained increases in the number of myosins in DRX *in vivo* may not induce hypertrophy, contrary to numerous HCM mutations. After long-term pressure overload of 12 weeks, cardiac function was dramatically preserved in a cMLCK overexpresser line, with respect to contractility and chamber size, interstitial fibrosis, and apoptosis (1). In these referenced studies and ours herein, increasing RLC-phosphorylation *in vivo* appears to blunt the initiation of hypertrophic response. Although not tested in our MYPT2-ablated mice, if the role of MYPT2 is limited to regulating cardiac MLCP activity, cardiac function at later time points after aortic banding are predicted to be comparable to that of cMLCK transgenic animals which showed continued preservation of normal cardiac function.

During the preparation of this manuscript, the phenotype of a MYPT2 conventional knockout mouse model was published by another group (58). This group used the C57Bl6/N background, which carries a loss of function of cMLCK caused by a SNP in the translation initiation site that generates an alternative start site with a frameshift (59). Indeed, there are several differences in the baseline phenotype attributable to the N-subtype, which has numerous unique mutations associated with heart failure, and differential responses to pressure overload (60, 61). MYPT2 KO partly rescued the baseline phosphorylation deficit in the C57Bl6/N background but did not rescue the marked reduction in heart weight (58). Our study using a conditional targeted approach in the adult C57Bl6/J subtype avoids mouse line–specific cardiac functional deficits and circumvents potential long-term gene ablation-induced compensatory changes. In our floxed animals, baseline cardiac RLC-phosphorylation was at normal levels, and we did not see a difference in heart size after tamoxifen-induced protein knockout in the cardiac myocytes.

Human mutations in cMLCK have been reported (62, 63), directly linking RLC-phosphorylation levels to heart failure. In a recent study using patient-derived iPSC-derived cardiomyocytes, a small molecule cMLCK activator was reported to alleviate contractile deficits caused by frameshift mutations in Mylk3 (64). Based on mouse models, in addition to the dedicated cMLCK, other kinases are expressed in the cardiomyocyte that contributes around 0.1 mol phosphate/mol RLC. Our studies herein show inhibition of MYPT2-regulated MLCP activity could increase RLC-phosphorylation even in hearts under stress, where cMLCK expression is reduced. The expression of MYPT2 protein is greatest in the heart and slow-twitch skeletal muscles, where increasing RLC-phosphorylation through transgenic overexpression of MLCKs had beneficial or no effects on baseline muscle contractility (1, 9, 65). The absence of MYPT2 in smooth muscles that make up the circulatory system and all hollow organs enhances the feasibility of systemic inhibition of MYPT2 to enhance RLC-phosphorylation. These data suggest MYPT2 as a tractable target for enhancing RLC-phosphorylation *in vivo*.

## Experimental procedures

### Ethical approval

Experiments were performed in accordance with the National Institutes of Health and Institutional Animal Care and Use Guidelines. The Institutional Animal Care and Use Committee at the University of Texas Southwestern Medical Center approved all procedures and protocols. Animals were sacrificed by the intraperitoneal administration of a lethal dose of tribromoethanol (250 mg/kg) for tissue collection.

### Generation of genetically modified mice

Mice containing floxed PPP1R12B alleles were generated in C57Bl6/J mice using CRISPR/Cas9 by the Transgenic Technology Core at UT Southwestern Medical Center. Guide RNAs were custom-designed by Sigma to insert LoxP sites flanking exon 1 of PPP1R12B, the gene that encodes MYPT2 protein. Correct insertion of LoxP sites was verified by sequencing. To reduce MYPT2 protein in adult mouse hearts, mice containing floxed alleles for PPP1R12B were crossed with a transgenic mouse line expressing a fusion protein of the Cre recombinase with the modified estrogen receptor binding domain (CreERT2) under the control of the cardiac-specific alpha-myosin heavy chain promoter (Mer-Cre-Mer) (66). Founder mice were bred and screened using custom designed primers and genotyping service by mousegenotype.com, and validated lines were maintained by genotyping service by Transnetyx. Mice (8–12 weeks old, mixed gender) were injected intraperitoneally with tamoxifen for 5 consecutive days at 1 mg/day doses. Hearts were collected 2 to 7 weeks later, as indicated, after the start of the tamoxifen injection. Mice did not show any visible signs of distress. All lines were kept in C57Bl6/J background, and inbred status was confirmed by genetic monitoring by Transnetyx.

### Pressure-overload hypertrophy model and echocardiography

Ten- to twelve-weeks-old male mice were subjected to TAC as described previously (67). Male mice were used for TAC because female C57Bl6/J mice have differential responses to TAC, that is beyond the scope of this limited study that focuses on biochemical contributions of myofilament proteins to baseline cardiac function (60, 68). An intraperitoneal injection of ketamine (100 mg/kg) and xylazine (5 mg/kg) was administered to anesthetize mice. The thoracic aorta was visualized through an incision in the left chest at the second intercostal space. A 27-gauge needle was laid across the thoracic aorta, an overlying ligature was imposed, and the needle was then withdrawn, resulting in vascular stenosis.

Nonanesthetized, lightly restrained mice were subjected to echocardiography using a Vevo 2100 system (VisualSonics) equipped with an MS400C scanhead, as previously described (69). M-mode recordings were acquired from a short-axis view at the level of the papillary muscles. When the largest and smallest left ventricular cavity areas were present, the left ventricular internal diameter (LVID) was measured at both the end of diastole (LVIDd) and the end of systole (LVIDs). The formula for calculating fractional shortening was (LVIDd-LVIDs)/LVIDd x 100%. Averages for each parameter, recorded at least three times, are provided.

### Preparation of heart extracts

Harvested hearts were excised free of atria, and ventricles were snap frozen within 1 min, using clamps prechilled in liquid nitrogen. Frozen ventricles were ground in liquid nitrogen to uniform heart powder, then stored in aliquots at −80 °C. For immunoblotting, an aliquot of heart powder (approximately 20 μg wet tissue weight) was simultaneously thawed and precipitated in 500 μl of 10% trichloroacetic acid and 10 mM dithiothreitol (DTT) to stop all enzymatic reactions. Precipitated tissue granules were rinsed with ethyl ether (3 times for 10 min each), exposed to air for a few minutes to evaporate the ether, and suspended in at least 30× volume (600 μl for 20 μg) of urea sample buffer containing 8 M urea, 20 mM Tris (pH 8.6), 23 mM glycine, 10 mM DTT, 4 mM EDTA, and 5% sucrose. Proteins were solubilized by extended continuous agitation on a digital Vortexmixer (Ohaus) set at 1400 rpm, adding urea crystals to saturation until samples appeared translucent (6 h at room temperature). Protein content was determined by Bradford assay (Bio-Rad) with bovine serum albumin as the standard. Samples were aliquoted and stored at −80 °C.

### Immunoblotting and antibodies

Tissue extract proteins solubilized in urea sample buffer were quantified by Bradford assay (Bio-Rad), then added to 0.25 volumes of 4xLDS sample buffer and reducing agent for SDS-PAGE per reagent instructions (Thermo Fisher). Protein solubilization quality and gel loading for immunoblots were predetermined by Coomassie-stained (Imperial Protein Stain, Thermo Fisher) gel image analysis after separation by 4 to 12% SDS-PAGE (Bolt, MOPS buffer system, Thermo Fisher). Separated proteins (3 μg/lane) were transferred onto a nitrocellulose membrane, then processed for immunoblotting using standard procedures (70). Briefly, membranes were incubated with specific antibodies diluted in 5% BSA/TBST overnight at 4 °C, washed in TBST 3 × 10 min, probed with horseradish peroxidase-conjugated secondary antibodies at room temperature for 1 h, washed in TBST 3 × 10 min, then developed using ECL Plus (Thermo Fisher), and imaged using Chemidoc MP (Bio-Rad). Antibodies used were: Phosphorylated MYPT1 (p696) from Millipore, pan MYPT from Abcam (#32519), and MYPT1 from Cell Signaling (2634s). Pan PP1c and PP1cβ antibodies were described previously (40).

### Measurement of RLC-phosphorylation

RLC-phosphorylation was measured by urea/glycerol-PAGE as previously described (19). Muscle proteins in an 8 M urea sample buffer were subjected to urea/glycerol-PAGE to separate nonphosphorylated from monophosphorylated RLC. Following electrophoresis, proteins were transferred to PVDF membranes, fixed in 0.4% glutaraldehyde/PBS for 5 min, washed in PBS for 3 × 5 min, and probed with a mouse monoclonal antibody against cardiac myosin (Enzo, F1093E1). The ratio of monophosphorylated RLC to total RLC (nonphosphorylated plus monophosphorylated) was determined by quantitative densitometry of developed immunoblots and expressed as mol phosphate per mol protein.

### Immunofluorescence microscopy

Hearts from adult male mice, 3 to 4 weeks post tamoxifen-injection, were retrograde perfusion-fixed through the aorta in 10% NBF, cryopreserved in 18% sucrose, and embedded in Tissue-Tek optimal cutting temperature compound for cryosectioning by the Histopathology core at UT Southwestern. For mouse cardiac myofibrils, mouse ventricles from 12week old adult mice were diced on ice into 1 mm long pieces, then homogenized using a 1 ml Dounce homogenizer using buffers and weight adjusted volumes previously described for porcine ventricular myofibrils by Solaro *et al* (71). Confocal imaging was performed in the Cell Biology and Imaging Core in the O'Brien Kidney Research Center, using a Zeiss LSM880 Airyscan laser scanning microscope equipped with Plan-Apochromat 10×/0.3 NA, 20×/0.8 NA, 25×/0.8 NA, and 63 × /1.40 NA oil-immersion objectives (ZEISS). Fluorescence images were acquired using ZEN black 2.3 software with a 20×/0.8 NA or 63×/1.40 NA objective, and Zeiss Immersion Oil 518F was used for 63×/1.40 NA, at constant room temperature. Acquired images were analyzed and regions of interest (ROIs) were further processed with ZEN 2.6 (blue edition) software. Reagents used were anti-Mypt2 from Proteintech 13366-1-AP (1:200), anti-cRLC from Enzo F1093E1 or from the Stull lab J545 (1:100), anti-ACTN2 from Abcam Ab68167 or Ab9465 (1:200), Phalloidin from Invitrogen R415 (1:2000), and Invitrogen prolong Gold with DAPI for mounting. All secondary fluorescent antibodies were from Thermo Fisher.

### Measure of cardiomyocyte cross-sectional area

WGA staining was performed by the Histo Pathology Core at UT Southwestern Medical Center. Briefly, paraffin-embedded sections were dewaxed, rehydrated, blocked in 1% BSA/5% Donkey serum in PBS, and then stained with Alexa Fluor 594-WGA (10 μg/ml, Invitrogen) and DAPI in blocking solution before mounting using anti-fade mounting media. Images of heart histology subjected to WGA were acquired on a Leica DMI 3000B fluorescence microscope at 20× objective magnification, imported into Image J v1.48 and cardiomyocyte cross-sectional areas were assessed by thresholded particle analysis. In brief, green-channel information from 8 bit TIFF images was converted to grayscale with RGB stack function. Individual cardiomyocytes were highlighted by setting thresholding for inclusion of intensity values between 0 (absolute black) and 56 (medium gray) to yield a differential between unstained cardiomyocyte cytoplasm and WGA-stained cardiomyocyte sarcolemma. Image J’s Particle Analysis macro was then used to count and obtain morphometric parameters in raw pixels with gating set for particles ranging 100 to 3000 pixels in area and with circularity between 0.5 and 1.0 to exclude capillaries and tangential cardiomyocytes.

### Assessment of SRX↔DRX equilibrium by mantATP chase assay

To measure the SRX state of myosin in skinned LVPM fibers from KO and control mice, nucleotide exchange experiments were performed, where fluorescent mantATP (Thermo Fisher Scientific) was replaced by nonfluorescent (dark) ATP using the IonOptix instrumentation as previously described (50, 56). Frozen hearts were thawed, and LVPM fibers were isolated in ice-cold pCa 8 solution (10^−8^ M Ca^2+^, 1 mM free Mg^2+^, total MgPr, propionate = 3.88 mM, 7 mM EGTA, 2.5 mM Mg-ATP^2−^, 20 mM MOPS pH 7.0, 15 mM creatine phosphate, and 15 U/ml of phosphocreatine kinase, ionic strength = 150 mM adjusted with KPr) containing 30 mM 2,3-butanedione monoxime, 15% glycerol, and 1 μM Calyculin A. After dissection to ∼2 × 0.5 mm size, muscle bundles were transferred to pCa 8 solution mixed with 50% glycerol (storage solution) and 0.5 μM Calyculin A and incubated for 1 h on ice. Then, the strips were transferred to fresh storage solution mixed with 1% Triton X-100 and 0.5 μM Calyculin A for ∼16 h at 4 °C. Chemically skinned muscle strips were placed in a storage solution and kept for up to 5 days at −20 °C until used for experiments. On the day of assay, small muscle strips of ∼1.4 mm in length and 100 μm in diameter were isolated from a batch of glycerinated skinned muscle bundles, treated with 1% Triton X-100 in pCa 8 buffer containing 5 nM microcystin for 30 min at room temperature, washed in pCa 8 buffer with 5 nM microcystin (3 times × 5 min) and then subjected to mantATP chase assays. The experiment was initiated by incubation of the fiber in a solution containing 250 μM mantATP in a rigor solution [120 mM KPr, 5 mM MgPr, 2.5 mM K_2_HPO_4_, 2.5 mM KH_2_PO_4_, 50 mM MOPS (pH 6.8), freshly prepared 2 mM DTT, and 5 nM microcystin]. After fluorescence intensity reached a stable level, the fiber was chased with 4 mM nonfluorescent ATP. Obtained fluorescence decay isotherms were fit to a two-exponential equation: Y = 1-P1[1-exp(-t/T1)]-P2[1-exp(-t/T2)], where P1 and P2 are the amplitudes of the fast phase and slow phase of decay, respectively, and T1 and T2—their respective lifetimes (in seconds). The proportion of myosin heads in the SRX and DRX states was calculated after correcting the fast phase of the fluorescence decay (P1) for the fast release of nonspecifically bound mantATP, as described earlier (50). Experimental fibers were saved in 10% TCA in water with 10 mM DTT and stored at −80 °C until used for protein gel electrophoresis.

### Statistical analyses

Data are expressed as mean ± SD. Statistical evaluation was carried out in GraphPad Prism. Unpaired two-tailed Student’s *t* test were used for two comparisons. For comparisons of male and female baseline values, or responses to surgery, significance was determined by two-way ordinary ANOVA followed by Tukey’s multiple comparisons post-test. Prism analysis output for each two-way ANOVA are provided in a supplemental Excel file. Specific tests used in comparisons and significance are described in respective figure legends.

## Data availability

All data generated or analyzed during this study are included in this article or are available from the corresponding author upon reasonable request.

## Supporting information

This article contains supporting information.

## Conflict of interest

The authors declare that they have no conflict of interest with the contents of this article.

## Supporting information

Supporting Information 2

Supporting Information revised
