# Supplementary material for: The MYPT2-regulated striated muscle-specific myosin light chain phosphatase limits cardiac myosin phosphorylation in vivo
Source: J Biol Chem. 2024 Jan 13;300(2):105652. doi: 10.1016/j.jbc.2024.105652 (PMC10851227; doi:10.1016/j.jbc.2024.105652)
Supplement: Supporting Information revised [file mmc2.docx]

Supporting Information

Table S1. Comparison of cardiac parameters measured by echocardiography.

|  | **PPP1R12B^f/f^/Cre-** | | | | **PPP1R12B^f/f^/Cre+** | | | |
| --- | --- | --- | --- | --- | --- | --- | --- | --- |
|  | Sham | | TAC | | Sham | | TAC | |
|  | Pre (N=6) | Post (N=6) | Pre (N=7) | Post (N=7) | Pre (N=5) | Post (N=5) | Pre (N=4) | Post (N=4) |
| LVIDd | 2.8 $\pm$ 0.1 | 2.7 $\pm$ 0.2 | 2.8 $\pm$ 0.2 | 2.7 $\pm$ 0.2 | 2.7 $\pm$ 0.1 | 2.9 $\pm$ 0.4 | 2.7 $\pm$ 0.5 | 2.6 $\pm$ 0.1 |
| LVIDs | 1.3 $\pm$ 0.2 | 1.3 $\pm$ 0.1 | 1.4 $\pm$ 0.3 | 1.4 $\pm$ 0.2 | 1.3 $\pm$ 0.2 | 1.5 $\pm$ 0.3 | 1.2 $\pm$ 0.2 | 1.3 $\pm$ 0.3 |
| %EF | 85 $\pm$ 5 | 85 $\pm$ 3 | 81 $\pm$ 7 | 83 $\pm$ 3 | 83 $\pm$ 4 | 82 $\pm$ 8 | 86 $\pm$ 4 | 88 $\pm$ 2 |
| %FS | 53 $\pm$ 6 | 53 $\pm$ 4 | 49 $\pm$ 7 | 51 $\pm$ 3 | 52 $\pm$ 6 | 50 $\pm$ 8 | 54 $\pm$ 5 | 56 $\pm$ 3 |
| %EF/wall thickness | 0.93 $\pm$ 0.1 | 0.90 $\pm$ 0.1 | 0.80 $\pm$ 0.1 | 0.90 $\pm0.2$ | 0.89 $\pm$ 0.1 | 0.93 $\pm$ 0.2 | 1.1 $\pm$ 0.2 | 0.94 $\pm0.2$ |
